# Electroencephalography in motor decision-making: a scoping review in athletes

**DOI:** 10.3389/fpsyg.2026.1794692

**Published:** 2026-04-24

**Authors:** Zidong Huang, Weiyue Xue, Andong Ding, Xiaozhuo Wei

**Affiliations:** 1 School of Physical Education, Jiangsu University of Science and Technology, Zhenjiang, China; 2 School of Physical Education, Yangzhou University, Yangzhou, China; 3 School of Naval Architecture & Ocean Engineering, Jiangsu University of Science and Technology, Zhenjiang, China

**Keywords:** cognitive hierarchy framework, electroencephalography, motor decision-making, neural efficiency, scoping review

## Abstract

**Introduction:**

The application of electroencephalography to investigate the neural mechanisms of motor decision-making in athletes is a rapidly evolving field. However, significant heterogeneity in experimental paradigms, neural metrics, and sample definitions currently hampers theoretical synthesis and practical translation.

**Methods:**

This study conducted a systematic scoping review following the PRISMA-ScR guidelines. Literature inclusion was strictly governed by the population-concept-context framework, focusing on athletic populations.

**Results:**

Based on 29 studies, we propose a three-level cognitive hierarchy model that abstracts motor decision-making into internal planning, external perception, and response inhibition. We show that expert advantage comes from dynamically contextually optimized neural resources, not a single “neural efficiency” profile. We also identify fundamental tension between ecological validity and methodological validity of the current evidence.

**Discussion:**

To bridge the gap from neural correlates to behavioral intervention, we further propose a bio-computational “assessment-to-intervention” pipeline. This pipeline integrates sensitive neural markers, computational cognitive modeling, and personalized intervention, aiming to provide an actionable framework for neuroscience-informed precision cognitive training in sports. This review not only offers a unified theoretical lens for understanding motor decision-making but also charts a concrete translational pathway for its future application.

## 1 Introduction

When players of opposing teams have the same physical fitness as well as technical and tactical skills, it is usually the athletes’ ability to make fast motor decisions that influence the outcome of the game. This is the case for athletes who can quickly perceive information, assess situations, and take the best action under pressure. Motor decision-making is not just a conditioned reflex but rather an intricate process embedded in many neural networks, including perception, cognitive analysis and sports selection (Wu et al., 2015; Gallivan et al., 2018; Khilkevich et al., 2024). In the field of sports science, it pertains to the cognitive process through which athletes select the optimal course of action based on perceived information within a dynamic and uncertain sporting context (Cotterill and Discombe, 2016; Tenenbaum and Filho, 2017; Pennock, 2020). Its fundamental purpose is to integrate information processing with behavioral selection, thereby impacting the ultimate outcomes of competition and enhancing athletic performance (Lorains et al., 2013; Cotterill and Discombe, 2016). For an extended period of time, research in this domain predominantly relies on the theoretical framework of information processing theory (Czyż, 2021). This theory conceptualizes the decision-making process as a sequence of cognitive stages and derives inferences from various behavioral indicators, including reaction time and accuracy (Czyż, 2021). In recent years, advancements in cognitive neuroscience have prompted researchers to concentrate on elucidating the real-time neural dynamic mechanisms underlying the decision-making process. Electroencephalography (EEG) offers a unique way to directly observe neural activity patterns during various stages of decision-making due to its excellent temporal resolution (Johnson, 2006, 2025). By analyzing the amplitude and latency of event-related potential (ERP) and the oscillation characteristics of EEG rhythm (such as α and β waves), EEG is capable of elucidating distinct cognitive phenomena, including early perceptual encoding and conflict detection, as well as the continuous allocation of neural resources throughout the decision-making process (Cortes et al., 2021; Saeidi et al., 2021; Sinha et al., 2023; Nazari et al., 2024).

Although research in this area has made considerable progress, this field still remains fragmented and methodologically heterogeneous. Different studies vary in balance between control of lab work and real sports situations, and few comparison studies are systematic. Different studies focus on different ERP components and analyses, making comparisons and integration difficult.

In light of this, the present study aims to employ a scoping review methodology and adhere to the PRISMA-ScR reporting guidelines (Tricco et al., 2018; Peters et al., 2021), seeks to systematically review and synthesize the existing literature on the application of EEG technology in the context of motor decision-making. This study concentrated on the motor decision-making process, which entails explicit behavioral choices, and sought to address the following central research questions: (1) What are the fundamental characteristics of research within this field? (2) Which experimental paradigms and EEG/ERP data acquisition and analysis methodologies were employed in the study? (3) What key neuroelectrophysiological aspects of motor decision-making were identified? (4) What are the limitations and controversies associated with the methodology and application of contemporary research? By answering the above questions, this study seeks to offer a methodological guide for future research and a theoretical foundation for developing neuroscience-based precise motor cognitive training programs.

## 2 Literature and methods

### 2.1 Defining the research question

The study was conducted following the guidelines outlined in the Joanna Briggs Institute (JBI) Scoping Review Methodological Manual (Pollock et al., 2022). Furthermore, the study protocol has been registered with the Open Science Framework (OSF) on 17 December 2025, and is publicly available at https://doi.org/10.17605/OSF.IO/YTFR8. Following a preliminary survey of the pertinent literature, the primary research questions were delineated, as outlined in the third paragraph of the introduction.

### 2.2 Literature inclusion and exclusion criteria

The PCC framework based on the scoping review, that is, the criteria are formulated according to the participants, concept and context.

Inclusion criteria: (1) Subjects (P): The target population was a class of athletes of different levels of competition. (2) Core concepts (C): EEG and ERP approaches are used to study motor decision-making for decision-making for behavioral choices or action plans. (3) Sports research conducted in labs, simulations, or real-world scenarios. Exclusion criteria: (1) Liquidities that are neither Chinese nor English (2) The types of literature include reviews, systematic reviews, meta-analyses, expert consensus documents, conferences abstracts. (3) Merely non-athletes and clinical patients were involved. (4) The study only reports resting state EEG or studies cognitive processes unrelated to decision making. (5) The full text of the document is unavailable.

### 2.3 Search strategy

This search was performed on PubMed, Web of Science, IEEE Xplore, Scopus, EMBASE, Cochrane Library, China National Knowledge Infrastructure (CNKI), Wanfang Data Knowledge Service Platform, Weipu Chinese Journal Service Platform, and other relevant Chinese and English databases. The search time is from the database creation till December 2025. The search consists of subject-specific terms and free-text keywords according to Boolean logic, with the formula modified according to the peculiarities of different databases.

CNKI is an example of Chinese search. The search method is (“electroencephalogram” + “EEG” + “event related potential” + “ERP”) and (“motor decision making” + “action selection” + “choice of exercise” + “tactical decision”) and (“sports” + “athletics” + “athlete” + “competitive sports”). PubMed served as an example for English search. The specific search method is (“sport“[Title/Abstract] OR “sports”[Title/Abstract] OR “athlete”[Title/Abstract] OR “player”[Title/Abstract] OR “players”[Title/Abstract] OR “tactical”[Title/Abstract] OR “motor“[Title/Abstract]) AND (“decision-making”[Title/Abstract] OR “decision-making”[Title/Abstract] OR “response selection”[Title/Abstract]) AND (“electroencephalography”[Title/Abstract] OR “EEG”[Title/Abstract] OR “ERP”[Title/Abstract]).

### 2.4 Literature screening and data extraction

The recovered articles were entered into Endnote 20.6 software, and duplicate records were deleted. Two researchers independently checked titles and abstracts, and excluded articles that were clearly not selected. The remainder of the literature was read completely before inclusion. The references of the included articles were manually reviewed for further studies. If the screening process is not agreed upon, another researcher will either arbitrate or facilitate consensus through discussion. Data extraction will be carried out using pre-designed standard forms, such as bibliography, neuroimaging measures, task specifications, and results.

### 2.5 Literature screening results

**Figure 1:**
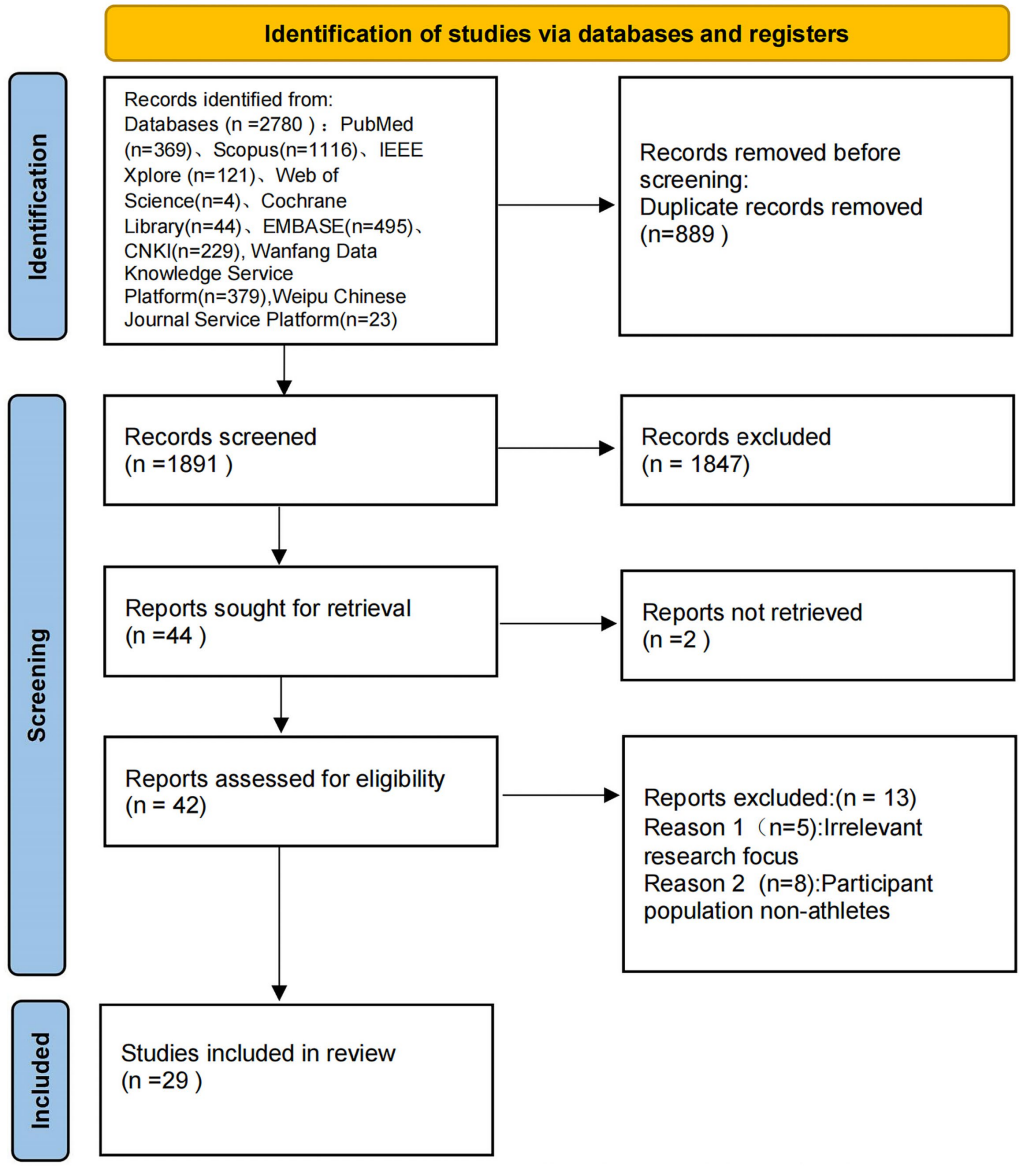
PRISMA flow diagram of the study selection process.

The search yielded 2,780 articles, 369 from PubMed, 1,116 from Scopus, 121 from IEEE Xplore, 4 from Web of Science, 44 from Cochrane Library, 495 from EMBase, 229 from CNKI. The results also included 379 articles from Wanfang Data Knowledge Service and 23 from Weipu Chinese Journal Service Platform. After removing duplicate articles, 1891 articles remain. After reviewing title and abstract, full text and references we selected 29 articles to be included in this study. The literature screening process is shown in Figure 1.

Basic characteristics of the included literature are shown in Table 1.

**Table 1:** Basic characteristics of included literature.

Sports	Author(s)	Country	Participants	Sample size	Research design and paradigm	Task settings	EEG metrics	Key findings
Basketball	Miao et al. (2025)	China	Elite basketball players	34 (ERP analysis of 30)	Experimental research: conflict decision-making	Basketball passing decision video task (conflict/non-conflict)	P2, P3b, N500, ERN	In conflict situations, decision-making becomes less accurate, reaction times increase, and confidence drops. ERP reveals increased amplitudes in P2, P3b, N500, and ERN, suggesting a multi-stage process for monitoring conflicts and detecting errors
	Chiu et al. (2020)	China	Elite basketball players (defender/forward)	46 (27/19)	Experimental study: Go /No-Go task	The visual Go/No-Go task	N2, P3	There was no behavioral difference between groups. ERP indicated that defenders had shorter N2 latency during Go tasks, longer N2 latency during No-Go tasks, and reduced P3 amplitude, suggesting location-specific neural efficiency
	Lucia et al. (2024)	Italy	Basketball players (experimental group/control group)	30	Experimental study: CMDT	CMDT: sprint, agility, decision making	BP, pN	In the CMDT group, sprint time decreased by 5.83%, agility improved by 3.55%, and decision reaction time improved by 10%. ERPs showed over a 50% increase in BP amplitude, suggesting enhanced motor preparation neuroplasticity
	Lucia et al. (2023a)	Italy	Semi-elite basketball players (female/male)	52 (28/24)	Experimental study: CMDT	CMDT vs. standard exercise training	BP, pN	The female CMDT group’s reaction time improved significantly, while the male group’s accuracy increased. ERPs indicated enhanced BP amplitude in females and pN amplitude in males, suggesting a compensatory effect specific to each sex
	Lucia et al. (2023b)	Italy	Semi-elite basketball players (female/male)	52 (28/24)	Experimental study: cognitive-motor dual task training	CMDT vs. standard exercise training	P1, N1 and P3	In the CMDT group, decision reaction time decreased by 5.4%, and accuracy improved by 25.8%. ERP results indicated a significant rise in P3 amplitude with no change in P1/N1, highlighting training-specific enhancements in late decision processing
	Lucia et al. (2023c)	Italy	Elite basketball players	30	Experimental study: multi-component training	Multitasking training (sprint, agility, decision-making)	P3	The experimental group showed a 13% faster sprint time, 14.2% better agility, and quicker decision-making, with enhanced P3 amplitude positively correlating with performance, underscoring cognitive-motor integration benefits
Badminton	Jin et al. (2010)	China	Badminton players (expert/novice)	36 (18/18)	Experimental study: action prediction	Landing position task	C1	Experts showed greater accuracy, with ERP revealing increased C1 amplitude and larger correct trial C1, suggesting plasticity in the early visual cortex
	Jin et al. (2011)	China	Badminton players (expert/novice)	36 (18/18)	Experimental study: action prediction	Landing position task (easy/difficult conditions)	P300, P2	Experts showed greater accuracy, with larger P300 and P2 amplitudes and shorter P300 latency, indicating optimized memory templates
	Xiong and Song (2024)	China	Badminton players (expert/novice)	24 (12/12)	Experimental study: action selection	Action selection task (return type judgment)	N100, P200, P300 and PSW	Experts exhibited greater accuracy with slower reaction times. ERP results indicated high N100 amplitude, low P200 amplitude, short P300 latency, and high PSW amplitude, signifying multidimensional neural dominance
Soccer	Fink et al. (2018)	Austria	Male soccer players (with over 10 years of athletic experience)	43	Experimental study: action observation and imagination	Video decision task (creative/regular)	α	Low α desynchronization in creative conditions suggests a focus on internal mental simulation, while highly creative individuals show significant left hemisphere α desynchronization linked to motor imagery
	Zhang et al. (2024)	Italy	Soccer referee (high anxiety group/low anxiety group)	70 (34/36)	Experimental study: Go /No-Go task	Penalty decision task (stress environment)	P300, N400	Judges with high anxiety showed larger P300/N400 amplitudes and lower accuracy under low pressure, but these differences vanished under high pressure, where they had shorter reaction times, indicating a stress-regulated anxiety effect
	Zhang et al. (2022)	China	Soccer referee (experienced group/physical education student group)	62 (30/32)	Experimental study: simulation decision	Home advantage scenario decision task	ERN	Home advantage boosts ERN amplitude, while the experienced group shows smaller ERNs and quicker, more accurate decisions, suggesting experience reduces interference
	Rubio-Morales et al. (2025)	Spain	Amateur soccer players	13	Experimental study: cognitive-body task	Stroop test, cycling, and combination task	IAPF, α	Cognitive and combination tasks led to greater psychological fatigue, as shown by EEG with decreased IAPF and increased α power, indicating lower brain readiness
Tennis	Costa et al. (2023)	Italy	Tennis athletes (expert/less expert)	37 (18/19)	Experimental study: space occlusion task	Space occlusion detection task	N1, pP1, pP2, pN2, P3	Performance was lowest when the torso and ball were hidden; expert P3/pN2 amplitudes increased, suggesting better sensorimotor integration, but 3D representation did not improve
	Wang and Liu (2018)	China	Tennis athletes (expert/novice)	60 (30/30)	Experimental study: situational decision-making task	General and specific situational decision-making tasks	P1, P2	Experts make quicker and more precise decisions in certain situations, as ERP indicates a small P1 amplitude and a large P2 amplitude in cognitive decision-making, optimizing intuitive choices
	Yi (2012)	China	Tennis athletes (expert/level 2/novice)	43 (15/13/15)	Experimental study-time-blocking technique	Task of predicting the trajectory of the ball	P1, N1, N200, P300, LNC, LPP	Experts show quick pre-hit reactions and high accuracy, with ERP indicating small early P1/N1 wave amplitudes and large late LNC/LPP wave amplitudes, highlighting dynamic resource allocation
E-sports	Gostilovich et al. (2023)	Russia	E-sports players (CS: Go) (expert/novice)	20 (10/10)	Experimental study: Oddball paradigm	Visual stimulus presentation; target counting	P300	Experts have quicker reaction times, with P300 latencies of 20–70 ms and amplitudes of 7–9 μV, showing rapid stimulus classification and efficient resource use
Fencer	Feng et al. (2010)	China	Fencer athletes (elite/lever-1/lever-2/non-level)	39 (8/14/11/6)	Experimental study: tactical decision	Continuous image tactical judgment task	P1, N1, N2, P3, PSW	The elite group exhibited greater accuracy and quicker decision-making, with ERP showing high P1/P3/PSW amplitudes and short PSW latency, reflecting strong exogenous attention and knowledge updating
Volleyball	Kanatschnig et al. (2025)	Austria	Volleyball athletes (expert/amateur/novice)	64 (16/26/22)	Experimental study: prediction setting	Predict volleyball settings task	θ, α, ERD/S	Experts showed high prediction accuracy. While θ synchronization and α desynchronization were strong, the differences between groups were not significant, partially supporting the neural efficiency hypothesis
Rubik’s cube	Zarei et al. (2025)	Denmark	Elite Rubik’s cube athletes	13	Experimental study on the execution of Rubik’s cube planning	Rubik’s Cube plans and executes tasks	δ, θ, α, β	Planning and executing EEG showed no difference. Executive performance correlated with δ activity in the occipital lobe (*r* = 0.71), while TOL correlated with θ activity in the temporal lobe (*r* = 0.67), indicating cognitive integration
Curling	Song et al. (2022)	China	Curling athletes (expert/novice)	60 (30/30)	Experimental study: time judgment	3D video duration judgment task	α, θ	Experts’ accuracy in specific situations was inversely related to throwing performance (*r* = −0.849), with EEG indicating elevated alpha and theta power
Baseball	Muraskin et al. (2015)	America	Baseball player (expert/novice)	19 (9/10)	Experimental study: Go /No-Go task	Task simulation baseball batting decision	CNV, α, SMA	The expert response is quicker with a low error rate. CNV showed greater amplitude and stronger SMA activation in No-Go trials, suggesting improved perception-action coupling
Table tennis	Yuhui et al. (2013)	China	High-level table tennis players (international master/lever-2)	18 (6/12)	Experimental study: serve rotation judgment	Serve rotation type judgment task	P700	International master athletes showed high accuracy, with ERP indicating short occipitotemporal conduction time, low P700 amplitude, and right brain dominance, reflecting neural efficiency
	Li-Bin et al. (2014)	China	Table tennis players (lever-2/novice)	30 (15/15)	Experimental study: Oddball paradigm	Single feature information judgment task	C1, P1, N1, P3	The expert group exhibited quick reaction times, with ERP indicating short latency and high amplitude of C1/P1, along with high amplitude of N1/P3, demonstrating experiential plasticity
	Zhiping et al. (2017)	China	Table tennis players (expert/novice)	28 (14/14)	Experimental study: simple/discriminatory response	Image recognition task	α, ERD/ERS	During the discrimination task, athletes showed significant alpha ERD in the frontal/central areas and minimal alpha ERD in the parietal/occipital regions, indicating selective neural efficiency
	Yan et al. (2026)	China	Table tennis players (the national level 2/novice)	55 (22/23)	Experimental study: delayed reminder task	Delayed prompt judgment task	Drift rate (v) and MVPA decoding	Athletes showed quicker and more precise rotation/direction judgment, with the hierarchical drift diffusion model (HDDM) indicating high v-values and MVPA decoding being early and stable, significantly involving the frontal region
Multiple sports events	Ramyarangsi et al. (2024)	Thailand	Female elite athletes (gymnastics/soccer/e-sports)	42 (14/14/14)	Experimental study: Oddball paradigm	Experimental study: Oddball paradigm	P300	Gymnastics shows the quickest response, soccer has low accuracy, and e-sports has a delayed P300 latency, indicating that different sports influence visual processing strategies, with e-sports demanding high cognitive effort
	Bianco et al. (2017)	Italy	Fencer/boxer/control group	39 (13/13/13)	Experimental study: visual Go/No-Go tasks	Visual inhibition control task	BP, pN, N1, P3	Players had quicker reaction times; fencers displayed larger pN/BP and N1/P3 amplitudes, whereas boxers had higher error rates, likely due to their unique athletic traits
	Diaz-Brage et al. (2018)	Spain/China	Basketball player /individual event athlete/game player	37 (13/12/12)	Experimental study: predict context task	Prediction sequence target detection task	P3b, α/β	There were no behavioral differences between groups; however, basketball players showed a larger P3b slope and stronger prefrontal-parietal connectivity during basketball conversations, indicating domain-specific processing

### 2.6 Document feature

In a diachronic analysis of the 29 studies we analysed we can see different stages of development. From the chronological point of view we see increased number of published articles since 2018, and nearly 50% of the total articles published between 2023–2025. This suggests motor neuroscience is rapidly growing as an interdisciplinary field. From the geographical point of view we see multipolar distribution, including China, USA, Austria and Italy.

As for sports, the distribution of research is quite concentrated. Ball games (23 articles, accounting for 79.3%), basketball (Chiu et al., 2020; Lucia et al., 2023a; Lucia et al., 2023b; Lucia et al., 2023c; Lucia et al., 2024; Miao et al., 2025); soccer (Fink et al., 2018; Zhang et al., 2022; Zhang et al., 2024; Rubio-Morales et al., 2025); badminton (Jin et al., 2010; Jin et al., 2011; Xiong and Song, 2024) these projects predominate in the field due to their dynamic and open characteristics. Emerging sports like e-sports (Gostilovich et al., 2023), Rubik’s cube (Zarei et al., 2025) this has also gained attention of researchers, and thus increasing the number of the number of research and also offering new dimensions of understanding the decision making process under different cognitive loads.

From the methodological point of view, the research domain has evolved from using the traditional experimental tasks, such as Oddball task and Go/No-Go task to specialized sports scenarios with high ecological validity. Techniques have evolved from studying individual ERP components to multi-modal fusion over time, frequency and space. While the focus is now on verifying basic neural efficiency to investigating neural plasticity produced by training. All these trends illustrate the evolution and logic of the research domain, provide evidence for classifying neural processes of motor decision making, and provide foundation for further analyses of methodological heterogeneity.

### 2.7 Classification of neural mechanisms based on cognitive hierarchy

Cognitive neuroscience research suggests that information processing in brain is dynamically interacted with two fundamental modes: top-down and bottom-up processing (Min and Park, 2010; Rauss and Pourtois, 2013; Xiong et al., 2023; Cheadle et al., 2024; Veale and Takahashi, 2024). Top-down processing is the process of expectations, knowledge, experience, context, perception and behavior. Bottom-up processing is the attention capture process driven by external sensory data. Based on this theory and keeping with the fundamental cognitive processes and neural indicators identified in the literature, we classify them into the following three categories: top-down control is mainly driven by internal planning, bottom-up processing is driven by external stimuli, and high-level control emerges from conflict between the two.

#### 2.7.1 Relying on internal planning and problem solving decision

Decision making is the highest level of control and is influenced by internal cognitive resources, mental simulation, working memory, and long-term sequence planning. It is a typical top-down control of the brain. Cognitive operations, which emphasize internal and sustained information processing, are associated with different neural patterns observed in frequency domains of EEG and functional connectivity. For instance, prefrontal θ oscillations are associated with working memory load and cognitive control, whereas parieto-occipital α oscillations have synchronization and desynchronization patterns that correspond to internal attentional focus and visual–spatial representation. These results show the neural benefits of expert athletes for offline cognitive processing. These studies studied the change in brain functional connectivity associated with reorganization and plasticity for complex dual task and multitask training of basketball players (Lucia et al., 2023a; Lucia et al., 2023c; Lucia et al., 2024). In the domain of soccer, scholarly investigations have examined the α wave activity in athletes’ brains during action observation and creative imagination, the mechanisms by which neural feedback enhances tactical cognition, and the neural activities that sustain cognitive effort under conditions of psychological fatigue (Fink et al., 2018; Rubio-Morales et al., 2025). The internal assessment of force and timing of curling athletes before precise stone delivery is correlated with distinct patterns of brain electrical rhythms (Song et al., 2022). During periods of rapid recovery, Rubik’s cube athletes exhibit enhanced neural characteristics that are indicative of efficient problem-solving and planning abilities (Zarei et al., 2025). The exceptional performance of e-sports players in classification tasks is correlated with distinct neural patterns related to attention control (Gostilovich et al., 2023).

#### 2.7.2 Perception and action decision based on external clues

This form of decision-making is the most intuitive process in movement, and decision making is based on fast external visual cues. The bottom-up processing properties are shown in the literature. In the 16 articles reviewed for this article, decision-making is made using external cues. However, the strength of experts is in their ability to facilitate and improve bottom-up processing using internal templates (top-down expectations) developed through intensive training. This means that bottom-up processing is regulated or filtered through a top-down process. So the basic cognitive needs are swift perceptual coding, pattern recognition and action anticipation. So the neurophysiological indices used in this article are used to describe processing sequence from C1, P1, and N1 component for early visual attention and feature extraction, P300 component for stimulus evaluation and cognitive resources, ERN or N500 component for response outcome monitoring. These studies are widely used in team ball sports, like football and basketball, and focus on athletes’ decision-making process for passing in conflict situations and their perceptual performance under dual-task situations (Lucia et al., 2023b; Miao et al., 2025). In net-based competitive sports such as table tennis, badminton and tennis, the main focus is on ball speed, spin, landing point and informational cues from an opponent’s actions. At present research paradigm typically utilizes rapid response tasks to short video stimuli (Jin et al., 2010; Jin et al., 2011; Yi, 2012; Yuhui et al., 2013; Li-Bin et al., 2014; Zhiping et al., 2017; Wang and Liu, 2018; Costa et al., 2023; Xiong and Song, 2024; Yan et al., 2026).

In addition, neural mechanisms that govern tactical decisions, offensive prediction, and ball classification have been explored for fencing, volleyball, and baseball (Feng et al., 2010; Kanatschnig et al., 2025). Some universities have implemented cross-project comparisons using Oddball and standard tasks like predictive context, in order to discover common neural substrates that govern rapid perception and decision making for the best athletes in different projects (Diaz-Brage et al., 2018; Ramyarangsi et al., 2024).

#### 2.7.3 Reaction inhibition and action selection decision

When external stimuli do not match internal task configurations, such as inhibitory responses or prepotent behaviors, decision-making becomes more complex. At this stage, cognitive control is activated in response to detected conflict. At this stage, processing is switched from regulated perception to active top-down control, which is used to resolve conflict and suppress responses. These neurophysiological markers indicate improved cognitive control functions. N2 is related to early conflict detection; P3 is related to inhibitory control and response selection; CNV is related to action preparation and anticipation; prefrontal theta band power observed in frequency domain analysis, which refers to the involvement of cognitive control networks. Cross-based studies often use established paradigms (Go/No-Go task) or simulate real decisions. In basketball research, we have seen differences in neural efficiency between athletes in different positions during inhibition tasks (Chiu et al., 2020). In the field of soccer research, attention has focused on decision making processes of referees under high pressure (Zhang et al., 2024) and neural mechanisms for error monitoring affected by home advantage (Zhang et al., 2022). The cognitive process underlying baseball batters’ decisions to refrain from swinging at crucial moments exemplifies the superior reaction inhibition capabilities characteristic of expert athletes (Muraskin et al., 2015). Moreover, cross-disciplinary research has substantiated the potential commonalities in the neural mechanisms underlying response inhibition among athletes engaged in direct-contact sports, such as fencing and boxing, utilizing standardized visual suppression control tasks (Bianco et al., 2017).

## 3 Discussion

### 3.1 The ecological validity paradox

One of the major problems of current work is that many of these tasks have been based on highly controlled, simplified laboratory models (e.g., Oddball, Go/No-Go) in order to build motor decision models. These reductionist tasks lack dynamic multimodal sensory complexity and time pressure that are inherent to real sports environments. Therefore, our proposed cognitive hierarchy (internal planning, external perception, response inhibition) represents the base cognitive structure of decision making rather than its fully embodied execution. While this approach isolates the core neural components, it limits immediate generalization of the framework to open-skill athletics. Translational research must bridge ecological gaps by validating these neural signatures (P300, N200) via mobile EEG and VR paradigms moving from static perceptual encoded to dynamic perception-action coupling.

### 3.2 Validation of the neural efficiency hypothesis

The neural efficiency hypothesis (NEH) is a theory that discusses how the brain functions when a person is mentally capable of solving a problem, and the brain processes information better. In recent years the focus of the theory has been expanded to sports in order to understand how the brain performs better during sport-specific tasks performed by elite athletes (Li and Smith, 2021). Research suggests that expert athletes experience adaptive neuroplastic changes as a result of prolonged professional training, leading to enhanced neural efficiency in task-specific activities (Guo et al., 2017; Li and Smith, 2021; Kanatschnig et al., 2025). Otherwise, however, the evidence is very complex and highly dependent on context, including both supporting results and numerous situations which need more interpretation. This complexity is associated with decision-making process rather than random. The cognitive hierarchy framework developed here provides a theoretical framework to explain this complexity and to explain the contradictory results in the different research of NEH.

For internally guided planning, “neural efficiency” is better seen as task-specific changes in brain rhythms (e.g., θ/α synchronization or desynchronization in task-relevant networks) rather than a global reduction in activation (Riddle et al., 2020). For example, in soccer simulations, synchronization of α rhythms in particular brain regions may suggest effective inhibition of irrelevant pathways. In working memory load, moderate increase in θ band power may suggest efficient and adaptive allocation of cognitive resources. The essence of efficiency is the ability of brains to precisely control the synchronization of internal neural clusters according to tasks, rather than general increase or decrease in overall neural activity. In fast perceptual motor decision making, expert athletes excel not only reduce activation, but optimize timing of their whole processing system. During the initial decision making, expert athletes often display increased wave amplitudes in early components, namely C1 and P1, in the primary visual cortex. This is not a cognitive deficit; rather, this is an adaptation of neural specialization. In the mid to late decisions, experts usually show reduced latency and increased amplitude of P300 component (related to stimulus evaluation and response selection). This implies that proficient perceptual encoding in the early stages conserves cognitive resources for later evaluative processes and thus faster reaction times and better overall neuroeconomic efficiency. This model of early specialization in exchange for delayed economic output is a major advance of neural efficiency hypothesis. As in response inhibition and action selection decision making expert advantage is functional specialization of cognitive control networks. Experts do not show generalized decrease of activation of brain regions. Rather they exhibit functional specialization of cognitive control networks, particularly prefrontal-anterior cingulate circuits. Rather than generalized global reduction in activation of cortical activation, expert athletes demonstrate highly special cognitive control networks. In response inhibition and action selection expert advantage is precise localised neural recruitment only in prefrontal-anterior cingulate circuits. Specifically, experts display transient but highly efficient activation patterns—manifesting as significantly enhanced amplitudes and shortened latencies in the N2 and P3 components. This dynamic signature signifies an optimized allocation of focal neural resources rather than a mere blanket attenuation of signal amplitude.

In summary, the expert advantage in motor decision-making is not limited to a single neuroeconomic model, but rather to a dynamic and multidimensional strategy of neural resource allocation. This strategy has varying properties depending on the basic cognitive processes involved in the decision-making task. Future work should focus on discovering different neural mechanisms involved in different decision-making processes and finding the optimal brain functions relevant to project execution.

### 3.3 Multidimensional review of methodological heterogeneity

A diachronic analysis of the literature shows that the EEG research on motor decision-making is markedly different. It is mainly driven by ecological approaches and multimodal analysis. This rapid change has led to a large methodological heterogeneity, due to the different strategies adopted by different teams to balance ecological validity and experimental control. This difference is evident in the following three aspects.

#### 3.3.1 Experimental paradigm field

Recent studies indicate that there is a large differences in experimental paradigms. Fifteen studies (51.7%) used highly simplified standard cognitive tasks (Oddball, Go/No-Go). The main advantage of these tasks is that they offer a robust control and cross group comparisons. Again, one of the limitations of these studies is that they tend to abstract and simplify the embodied complexity of real motor decision making, which raises doubts on ecological validity. As Gostilovich et al. (2023) showed, the neural dominance observed in e-sports experts is task specific. This suggests that neural mechanisms found in simplified experimental tasks may not directly apply to real world competition. In contrast, 14 studies (48.3%) used motor decision-making tasks with high ecological validity in real world decision making environments. These paradigms can reproduce real world scenarios, but lack standard parameters such as trial sequence ratios, video duration, and response requirements which are not uniform and therefore cannot be easily comparison between groups.

#### 3.3.2 Selection of neurophysiological indices

Substantial heterogeneity also exists in the selection and analysis of neurophysiological indices. First, there are some differences in the selection and analysis of neurophysiological indices. Although 51.7% of studies (15 articles) focused on the P300 component (late decision making), only 27.5% (8 articles) focused on components (C1, P1, N1) related to early perceptual encoding. This “late heavy, early light” selection bias may result in incomplete temporal understanding of neural mechanisms that underlie expert advantages (in particular early perceptual optimization) of the neural mechanisms that underlie expert advantage. Secondly, there are significant differences between studies concerning key analytical parameters for the same ERP component (defining the temporal window definition, baseline correction, and selection of measurement electrode sites). These technical differences are directly affect the comparability of research results. Thirdly, frequency domain analysis can be a useful complement to ERP research but is not without its challenges, especially regarding inconsistent frequency band division standards and variability quantization methods. For example, while both Kanatschnig et al. (2025) and Zarei et al. (2025) used frequency domain analysis, the different definition of the band used in each study made it difficult to integrate their results cross-study integration of their results, and the variability in EEG acquisition parameters (e.g., sampling rates, filter settings, processing workflows) which are not classified as features in this study, further impedes comparability and reproducibility.

#### 3.3.3 Study design and sample

The variation in study design and sample size further breaks apart the field. There are still four articles had sample sizes below 20 participants. Also, the definition of an “expert” athlete varied greatly from studies with world-class competitors to second-tier athletes, making direct comparisons between expert-novice models difficult.

#### 3.3.4 Implications for evidence strength and the proposed framework

Collectively, the methodological differences described above (paradigm divergence, analytic variability, sample variability) constitute the strength of evidence supporting our cognitive hierarchy framework. Scoring review does not require risk-of-bias appraisal. Underpowered studies (*n* < 20) increase the risk of false-positive neuroplastic effects. Also, inconsistent operationalization of “expertise” and the use of simplified paradigms make it difficult to generalize specific neural efficiency signatures cross-study. Therefore, framework and neural correlates present here should be interpreted as a robust organizing heuristic and a set of falsifiable working hypotheses, rather than as definitive biomarkers. Solidifying the framework requires future large scale preregistered studies using standard psychometric baselines, consensus definitions of expertise, and more balance between experimental control and ecological validity.

### 3.4 Practical implications and future transformation

**Figure 2:**
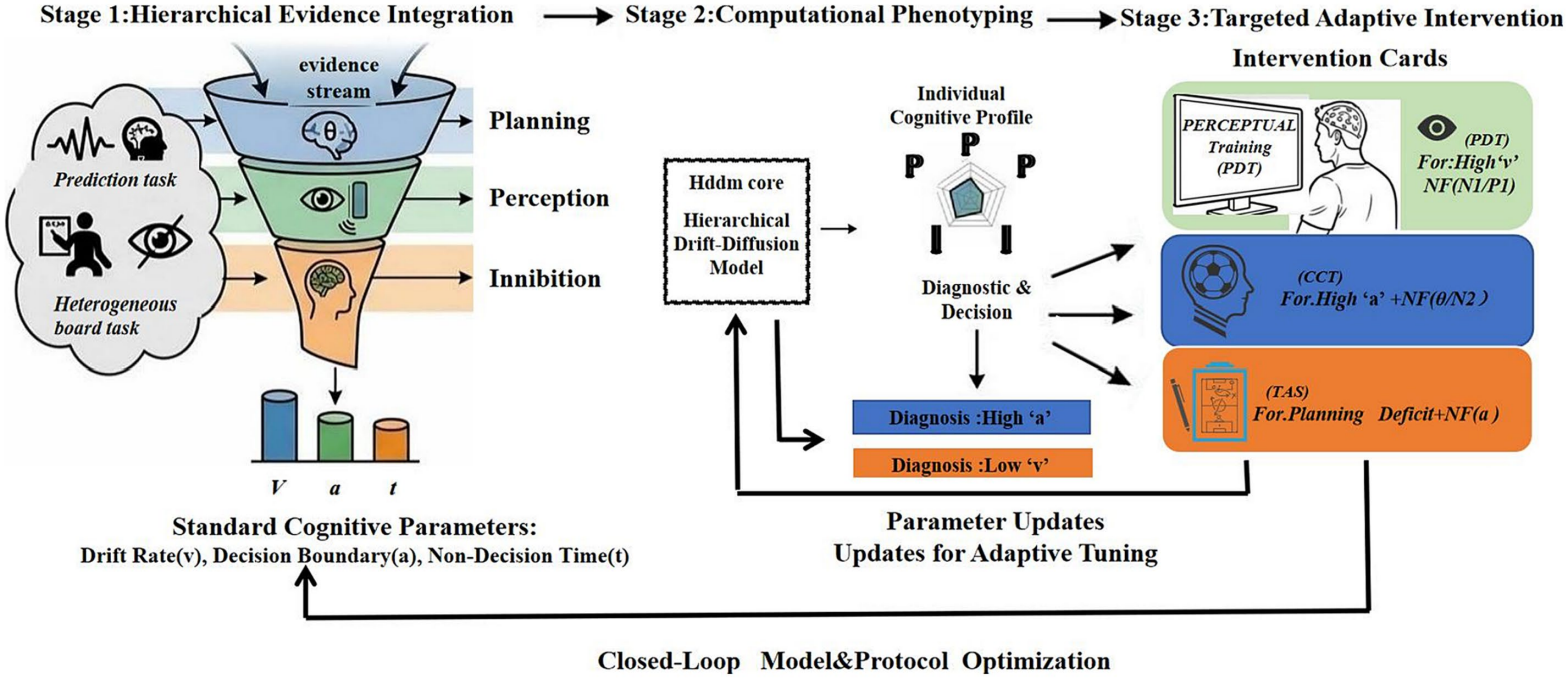
A closed-loop bio-computational pipeline for personalized motor decision training.

The previous discussions highlight the challenge of bridging the gap between the “neural correlates” and the “behavioral intervention.” The three main challenges identified—the paradox of ecological validity, context-dependent of the neural, and significant methodological heterogeneity—show that no single neural marker is directly and robustly applied. These challenges agree on one basic need: the need for a standard systematic pathway which integrates multilevel neurocognitive evidence into personalized, actionable training. To this end we propose bio-computational “assessment-to-intervention” pipeline (Figure 2), designed not only as an additional suggestion, but as an “ultimate solution” to the fragmentation discussed above.

#### 3.4.1 Multidimensional baseline bio-profiling

First, we need to define the athlete’s cognitive-neural functions. Based on the results of this review, we should start with EEG markers that are sensitive to training and aligned with our cognitive hierarchy: (1) Stimulus evaluation efficacy: quantify late-stage decision efficiency using P300 amplitude and latency in Oddball or Go/No-Go models. (2) Executive control and cognitive load: measure frontal theta oscillations during conflict resolution or sustained attention tasks as a consequence of top-down resource mobilisation. (3) Early perceptual encoding: Record early components such as N1/P1 during sport-specific anticipatory tasks to assess perceptual expertise and attentional allocation. Foundational studies using controlled paradigms have elucidated the basic neural mechanisms of rapid stimulus discrimination (Sherwin et al., 2012). Referencing this fundamental knowledge is crucial for interpreting how expert athletes optimize these early sensory processes within complex, sport-specific contexts.

#### 3.4.2 Integration of computational cognitive modeling

To be able to make more than a neural correlation and to learn how the behavior variance is encoded in computation, such biomarkers must be merged with computational models such as the HDDM. This bridges the gap between neural processes (e.g., reaction time, accuracy) and cognitive-computational methods (e.g., decision caution, information accumulation efficiency). Computational models decompose this black box, and can better map specific EEG patterns to computational parameters which govern decision behavior. For example, the drift rate (which is the speed of information accumulation) and decision boundary (which is the response caution). Notably, computational neuroscience work in non-athlete populations has demonstrated the feasibility of this approach. For instance, Wang and Zhang (2025) linked specific neural dynamics to drift rate variations in a perceptual decision task, providing a direct blueprint for how neural activity can be formalized as a computational decision parameter. The key benefit of this integration is its mechanistic interpretability. For example, it can determine whether a delayed P300 latency is driven by evidence accumulation deficit (low drift rate) or an overly conservative decision threshold (high boundary), so that interventions are a target for the computation-level interventions.

#### 3.4.3 Tailored intervention prescription and implementation

Based on the dual “bio-computational” profile, targeted intervention can be implemented: If an athlete has delayed P300 latencies and drift rate under pressure under pressure, training would focus on early sensory encoding and evidence accumulation, and stimulus-locked closed loop neurofeedback training would focus on specific frequency bands or early ERP components. If the bottleneck is an excessively cautious decision boundary, with high frontal theta activity, training should focus on rapid decision making under pressure, with neurofeedback training for executive control recruitment efficiency.

#### 3.4.4 Empirical validation and model iteration

The effectiveness of the intervention is measured by comparing the pre- and posttest performance on different set of metrics (behavioural data, ERPs, HDDM parameters, oscillation power). This validation confirms the effectiveness of individual trainings and provides a database that can be used to continuously optimize the pipeline and to build a robust evidence-based “assessment-intervention-reassessment” loop.

This framework directly addresses the fragmentation identified in the current literature. Rather than replacing existing sport-specific training, it offers a meta-framework that synthesizes disparate neural markers, heterogeneous experimental paradigms, and diverse computational models into a coherent system focused on resolving specific cognitive deficits.

We are reminded that the proposed “assessment-to-intervention” pipeline is a top level theory and proposal (working hypothesis) that needs future validation and thus provides a falsifiable and actionable roadmap. Validation involves first feasibility studies in particular sports, then mechanistic experiments to verify the proposed EEG-computation-behavior link, and finally random controlled trials to test the efficacy against the current intervention methods.

## 4 Conclusion

This scoping review summarized 29 studies to address the gap in understanding the neural basis of motor decision-making in athletes. The main contribution of this work is the proposed three-level cognitive hierarchy (rapid perception-action, response inhibition/selection, internal planning) reconciling differing results, suggesting expert advantage is a dynamical and context-dependent optimization of neural resources, not a single “neural efficiency” profile.

We have assessed the evidence supporting this framework in light of the fact that construction by simple laboratory tasks makes it a model of core cognitive structure, which can be fully expressed in ecologically valid settings, and the current strength of evidence is due to methodological differences, so we present it as a set of working hypotheses which can be refined by more standard large scale studies.

Most importantly, to catalyze translational progress, we propose a concrete bio-computational “assessment-to-intervention” pipeline. This roadmap—moving from multidimensional neural profiling and computational modeling to personalized intervention and validation—provides a direct pathway to transform the theoretical insights of this review into actionable protocols for cognitive enhancement in sports.

In summary, this work advances the field by providing a unifying theoretical framework, a clear-eyed assessment of the evidence, and an innovative blueprint for turning neuroscience into practice, thereby shifting the study of motor decision-making from descriptive correlation toward mechanistic explanation and precise application.

## Data Availability

The original contributions presented in the study are included in the article/supplementary material, further inquiries can be directed to the corresponding author.
